# Ogilvie's syndrome with caecal perforation after Caesarean section: a case report

**DOI:** 10.4076/1752-1947-3-6177

**Published:** 2009-06-05

**Authors:** Arin K Saha, Eleanor Newman, Matthew Giles, Kieran Horgan

**Affiliations:** 1 Department of Surgery, The General Infirmary at Leeds, Leeds Teaching Hospitals NHS Trust, Great George Street, Leeds, LS1 3EX, UK

## Abstract

**Introduction:**

Ogilvie's syndrome describes the phenomenon of an acute colonic pseudo-obstruction without a mechanical cause. It is rare but has been reported to occur after Caesarean section. It can lead to bowel perforation or ischaemia.

**Case presentation:**

A healthy, 28-year-old Caucasian woman presented 2 weeks past her expected date of delivery for her first pregnancy. She underwent an uncomplicated elective Caesarean section but developed abdominal pain and bloating postoperatively and was subsequently diagnosed with acute colonic pseudo-obstruction, also known as Ogilvie's syndrome.

**Conclusion:**

This case report highlights the rare, but potentially dangerous, diagnosis of Ogilvie's syndrome after Caesarean section. It is of particular interest to obstetricians, midwifery staff and general surgeons and shows the importance of accurate diagnosis, regular abdominal reassessment and early senior input to ensure appropriate and rapid treatment.

## Introduction

Ogilvie's syndrome (OS) was first described in 1948 [1] and is an acute colonic pseudo-obstruction without a mechanical cause. The case reported here describes a patient who developed OS with caecal perforation after a caesarean section.

## Case presentation

A healthy, 28-year-old Caucasian woman in her first pregnancy (gravida 1, parity 0) with grade one placenta previa presented 2 weeks past her expected date of delivery (at 39+2/40) for elective Caesarean section. She had had no pre-operative treatment for her condition and Caesarean section was carried out under spinal anaesthesia (2.7ml 0.5% bupivicaine with 300mcg; L3-L4 intervertebral space). There were no operative complications and there was minimal maternal blood loss. The total operating time was 11 minutes and a healthy baby boy weighing 3.1kg was delivered.

**Figure 1:**
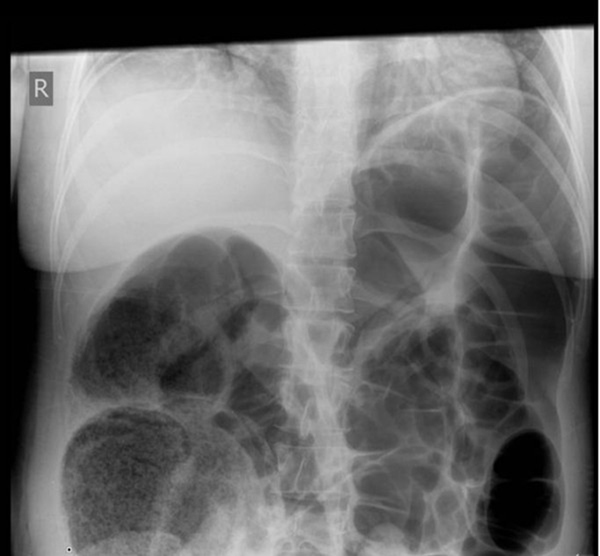
**Plain supine abdominal radiograph showing widespread colonic dilatation**.

Postoperatively, the patient initially recovered well, although she required paracetamol (1g 4 × daily), diclofenac (50mg 3 × daily) and dihydrocodeine (60mg 4 × daily) regularly for analgesia. On postoperative day (POD) 1, she reported abdominal distension and absolute constipation but no abdominal pain, and she was treated with lactulose. She developed shoulder-tip pain in the early hours of POD 2 which developed into colicky, central abdominal pain although she had passed flatus and had no vomiting. Physical examination revealed soft, abdominal distension with right-sided abdominal tenderness and no clinical evidence of peritonitis. Plain radiographs revealed widespread colonic dilatation with no free air (Figure 1) and a provisional diagnosis of acute colonic pseudo-obstruction (OS) was made.

**Figure 2:**
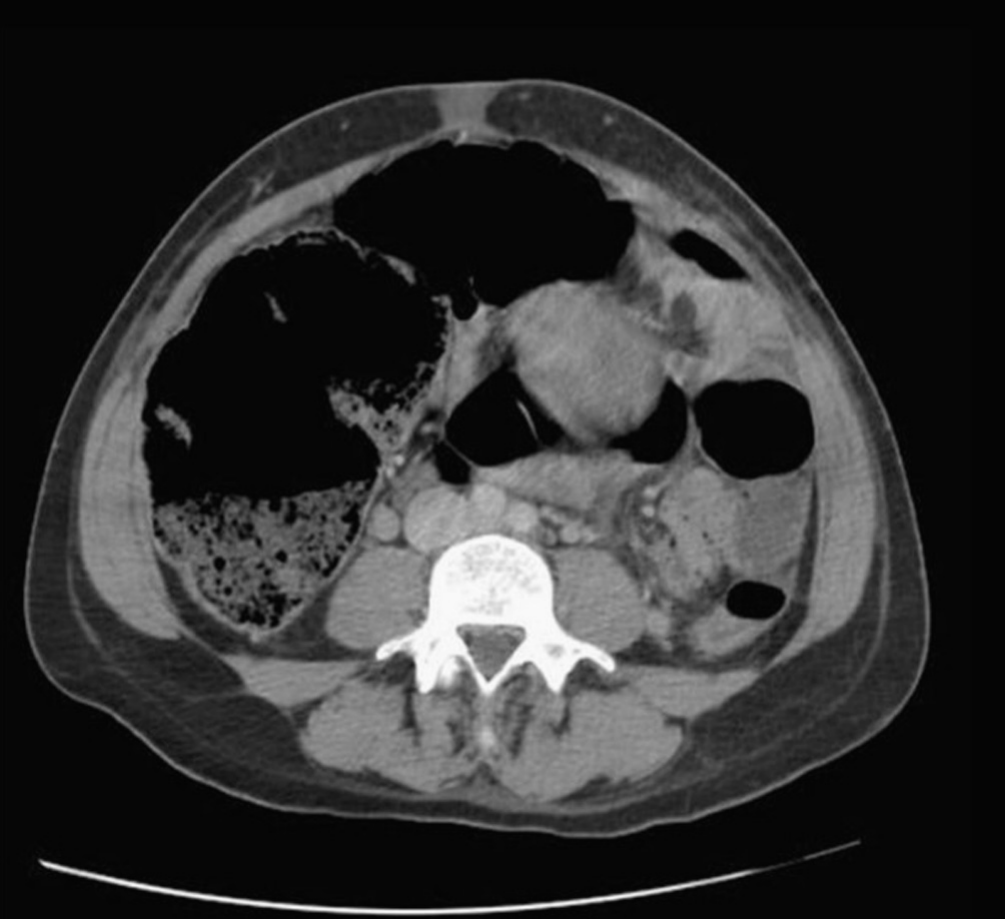
**Abdominal computed tomography image showing marked caecal dilatation**.

Conservative management which comprised nasogastric tube insertion, intravenous fluids and analgesia was instituted. Over POD 2, the patient's condition did not improve and two 2mg doses of intravenous neostigmine were given prior to attempted colonoscopic decompression. A rectal tube was inserted after colonoscopic decompression, although the patient did not have any symptomatic benefit. Despite these interventions, her condition deteriorated and her right-iliac fossa pain worsened. Computed tomography (CT) imaging showed widespread colonic dilatation and a maximum caecal diameter of 8cm (Figure 2).

The patient proceeded to emergency laparotomy where a significantly distended colon with caecal perforation and evidence of caecal ischaemia was found. There was no evidence of mechanical obstruction. A right hemicolectomy was performed with primary two-layer seromuscular anastomosis. Postoperative recovery was unremarkable.

## Discussion

Acute colonic pseudo-obstruction (or OS) is rare and has been reported as isolated case reports or small case series. It is described by a clinical and radiological picture of acute large bowel obstruction without a mechanical cause. It has most commonly been reported after pregnancy or Caesarean section, although has also been reported to occur after trauma and severe burns.

The pathophysiology of the condition is still unclear although one explanation is that an imbalance between sympathetic and parasympathetic innervation to the colon results in an overall excess in sympathetic activity [2]. This explanation is given credence by the location of autonomic nerves close to structures at risk during Caesarean section, including the cervix and the vagina. However, it is likely that the true pathogenesis is multifactorial.

Management of OS can be classified into non-surgical and surgical treatment, although arguably diagnosis and recognition are the most important aspects of care. Indeed, significant morbidity and mortality from the condition has been reported when there is a wrong diagnosis, most commonly paralytic ileus, and a delay in the diagnosis of bowel perforation, usually by junior obstetric or general surgical doctors [3].

Progressive abdominal distension is often painless at first. Importantly, bowel sounds are usually present and may be normal. Initial management should include intravenous fluid therapy, nasogastric suction and a plain abdominal X-ray. Most cases do not require surgical intervention and settle with either endoscopic or pharmacological therapy. Colonoscopic decompression is successful in the majority of cases, although recurrence is common. Pharmacological treatment includes naloxone, cholinergic stimulation with neostigmine or erythromycin and cisapride. In our patient, both colonoscopic decompression and neostigmine failed to resolve the pseudo-obstruction, and she developed progressive caecal distension and subsequently caecal perforation and peritonitis. Surgical treatment is indicated when the caecal diameter is greater than 9cm or there is evidence of perforation and comprises either caecostomy or, if ischaemic bowel is present, limited right hemicolectomy with or without primary anastomosis.

## Conclusion

The authors believe that OS, though uncommon, is a diagnosis to consider when investigating patients who have recently undergone Caesarean section. We advocate early general surgical referral and draw attention to the importance of regular abdominal examination to assess response to treatment and determine when surgical intervention is needed. Furthermore, we urge early escalation to senior obstetric and general surgical opinion if concerns persist.

## Abbreviations

CT: computed tomography; mcg: micrograms; OS: Ogilvie's Syndrome; POD: post-operative day.

## Consent

Written informed consent was obtained from the patient for publication of this case report and accompanying images. A copy of the written consent is available for review by the Editor-in-Chief of this journal.

## Competing interests

The authors declare that they have no competing interests.

## Authors' contributions

AS was a major contributor in writing the manuscript and interpreted the data regarding the operation and outcomes. EN gathered and analysed the data regarding the obstetric history and the non-operative management of the patient; she was also a major contributor in writing the manuscript. MG performed the general surgical operation and provided the data and details of the operation. KH was the consultant surgeon in charge of the patient and provided the intellectual basis for the report; in addition, he was a major contributor to the discussion. All authors read and approved the final manuscript.
